# Hidden Coherence Bursts and Time-Tagged Holevo Fragment Information Under Finite-Resolution Observation in a Central-Spin Environment

**DOI:** 10.3390/e28080842

**Published:** 2026-07-29

**Authors:** David Rebollo-Martínez, Manuel Rebollo-Salas

**Affiliations:** 1 Master in Quantum Technologies, Universidad Internacional Menéndez Pelayo (UIMP), 28040 Madrid, Spain; 2 Department of Physiotherapy, University of Seville, 41009 Seville, Spain

**Keywords:** temporal coarse-graining, open quantum systems, decoherence, Quantum Darwinism, central spin model, spin bath, Holevo information, hidden coherence bursts, finite-resolution observation, NV centers

## Abstract

We study a structured QND central-spin testbed under a hybrid finite-resolution protocol. The central-spin state is temporally integrated without retaining the internal readout-time label, whereas environmental fragment information is quantified by a time-tagged scalar-averaged Holevo benchmark. On a converged numerical grid, we identify finite time regions in which the integrated central-spin state has low off-pointer visibility while model-resolved late-time coherence bursts remain present and the typical-fragment Holevo benchmark exceeds a prescribed threshold. A detector-window variance decomposition supplies a model-assisted diagnostic; it requires a fine-grained trajectory or a microscopic model. The Holevo quantity is an upper bound on accessible classical information, and no fragment measurement attaining it is constructed. Grid refinement, an independent trapezoidal quadrature, and a half-cell grid shift preserve the qualitative four-region baseline structure. A sampled-subset calculation benchmarks the typical-fragment classification, but we do not establish constructively disjoint or state-integrated fragment redundancy. A single-realization detector-width/disorder map and a 100-realization ensemble delimit the structured mesoscopic scope. The dimensional conversion is an NV-like scale estimate for an engineered quasi-homogeneous testbed and not an experimental protocol for an arbitrary natural bath.

## 1. Introduction

Decoherence theory explains why selected observables of an open quantum system become effectively classical: Environmental monitoring suppresses interference between dynamically stable alternatives and selects a pointer algebra [1,2,3]. Quantum Darwinism and related objectivity frameworks add a spatial-information layer: classical records become objective-like when many fragments of the environment redundantly encode the same pointer information [4,5,6,7,8]. Structured environments were already shown to modify how redundancy is built and accessed [9]. Subsequent work has made this language more precise: Ordinary Quantum-Darwinist signatures, strong Quantum Darwinism, and Spectrum Broadcast Structure are related but not equivalent, and a plateau of total mutual information alone is not a sufficient certificate of strong objectivity [10,11,12]. This motivates the conservative Holevo-based terminology used throughout the present manuscript.

The question addressed here is deliberately finite-resolution. We do not claim that classical continuity is a universal primitive, nor that microscopic dynamics literally disappears and reappears. We ask a narrower question: When can a temporally integrated system state have low off-pointer visibility while a separately time-tagged fragment-information benchmark remains high and faster recoherence structure is present in the microscopic model? This is a hybrid two-channel construction and not the response of one detector applied identically to both system and fragments. The scalable fragment values are typical-fragment estimates and not constructed counts of simultaneous disjoint fragments.

Recent theoretical and experimental developments sharpen this motivation. Models of amplification show how Holevo information can track the effective classical channel between a system and environmental fragments in structured settings [13], while Hamiltonian classifications show that pointer-compatible, approximately separable interactions favor Quantum Darwinism and that intra-environment scrambling competes with the build-up of objectivity [14,15]. Spin-environment and laboratory studies then show how redundancy can be generated, degraded, or observed in concrete settings, from spin baths and nitrogen-vacancy centers to photonic and superconducting platforms [16,17,18,19,20].

This framing is motivated primarily by quantum objectivity and by central-spin physics. Spin-bath models provide a controlled setting in which a central degree of freedom is monitored by many environmental spins [21,22,23,24,25]. Structured spin environments also show that the operational definition of a fragment can decide whether redundancy appears at all [26], and realistic interacting or dissipative spin baths can alter central-spin coherence in nontrivial ways [27]. Ultrafast driven systems, including high-harmonic generation in solids, play only a secondary role: They illustrate that observed dephasing times can differ sharply from naive microscopic timescales and can involve collective radiative effects [28,29,30,31,32,33,34], but they do not validate the present spin-bath mechanism. Phase-resolved free-electron interferometry shows how finer temporal and phase resolution can reveal sub-cycle structure hidden from aggregated readouts [35,36]; ultrafast electron microscopy provides a related high-resolution analogy [37]. These are methodological analogies and not experimental validations of the central-spin construction.

The central model is a scalable central-spin fragment-information model. The interaction Hamiltonian selects the pointer basis, the fine-grained coherence contains recurrent late-time bursts beyond an operational Gaussian-envelope cutoff, and the bath spins define fragment Holevo quantities. Temporal integration can suppress the system’s coherence through cancellation. Because the model is QND, it describes a stable pointer distribution rather than a changing classical trajectory; a weak non-QND control tests only the sensitivity of reduced-state signatures to small pointer-population dynamics.

The distinction between retained and discarded readout information follows standard quantum-measurement theory [38], while recent first-principles work on emergent decoherent histories provides complementary context for classical-history formation under coarse-grained observation [39].

This article makes three contributions. First, it defines a hybrid finite-resolution protocol and gives the detector-window variance decomposition used to diagnose hidden fine-grained coherence. Second, it solves a central-spin model, reports converged numerical qualifying regions, and compares typical-fragment and sampled-fragment Holevo benchmarks together with detector-kernel, disorder, threshold, and weak non-QND controls; non-Markovianity motivates treating bath structure as part of the mechanism rather than as a nuisance parameter [40,41,42]. Third, it provides an NV-like dimensional scale estimate and states the QND, measurement, state-integrated, and scaling limitations explicitly.

## 2. Operational Framework

Before giving the formal definitions, we fix the operational meaning of the symbols. The random variable *X* denotes the pointer value of the system. A fragment *F* is a subset of environmental degrees of freedom used to define information about *X*. The quantity $D_{\mathrm{cl}}$ measures residual off-pointer visibility, $D_{\mathrm{cont}}$ measures how much the integrated pointer probabilities change over the observational horizon, and $I_{\mathrm{burst}}$ records whether fine-grained coherence is present inside the same time window. Fragment information is summarized by two explicitly limited quantities: $R_{\delta ,\mathrm{typ}}^{\Delta }$ is a scalable typical-fragment estimate, and $R_{\delta ,\mathrm{samp}}^{\Delta }$ is a sampled-subset estimate. Neither is a constructively verified count of simultaneous pairwise-disjoint fragments.

For the central-spin setting studied below, let $H_{S}≃C^{2}$ and let $P_{x}=|x\rangle \;\langle x|$, x∈{0,1}, be the rank-one pointer projectors selected by the system–environment coupling. The associated commutative pointer algebra is

(1) $$A_{★}=\left\{\sum_{x}a_{x}P_{x}\right\}.$$

The pinching map onto this diagonal pointer algebra is

(2) $$\Pi_{★}\left(\rho \right)=\sum_{x}P_{x}\rho P_{x},\;Q_{★}=I-\Pi_{★}.$$

Finite-resolution observation is represented by a causal normalized kernel $w_{\Delta }\left(s\right)$ supported on 0≤s≤Δ. We use a boxcar kernel for the main figures and compare it with truncated exponential and one-sided Gaussian kernels in the robustness analysis, following the standard finite-bandwidth logic of continuous measurement and filtering [38]. Production integrals use a composite midpoint rule. With M=Δ/Δt and $s_{j}=\left(j+1/2\right)\Delta t$,

(3) $${\langle f\rangle }_{\Delta }^{\mathrm{mid}}\left(t\right)=\frac{\sum_{j=0}^{M-1}w_{\Delta }\left(s_{j}\right)f\left(t-s_{j}\right)}{\sum_{j=0}^{M-1}w_{\Delta }\left(s_{j}\right)}.$$

An independently implemented composite trapezoidal rule, including both endpoints with half weights, is used as a quadrature control. The two rules are compared under successive grid refinement before numerical region boundaries are reported. The system channel is

(4) $$E_{S}^{\Delta }\left[\rho_{S}\right]\left(t\right)={\bar{\rho }}_{\Delta }\left(t\right)=\int_{0}^{\Delta }w_{\Delta }\left(s\right)\rho_{S}\left(t-s\right)\;ds.$$

This channel discards the internal readout-time label. It can therefore suppress the observed off-pointer component by cancellation between complex coherence amplitudes at different times. The operational classicality defect is the trace distance from the pointer algebra using the standard factor 1/2:

(5) $$D_{\mathrm{cl}}\left(t;\Delta \right)=\frac{1}{2}{∥{\bar{\rho }}_{\Delta }\left(t\right)-\Pi_{★}{\bar{\rho }}_{\Delta }\left(t\right)∥}_{1}.$$

The entropic defect is the entropy generated by pinching, equivalently the relative-entropy coherence associated with the pointer-algebra projection [43],

(6) $$E_{\mathrm{cl}}\left(t;\Delta \right)=S\;\left(\Pi_{★}{\bar{\rho }}_{\Delta }\left(t\right)\right)-S\;\left({\bar{\rho }}_{\Delta }\left(t\right)\right)=D\;\left({\bar{\rho }}_{\Delta }\left(t\right)∥\Pi_{★}{\bar{\rho }}_{\Delta }\left(t\right)\right),$$

with all Shannon and von Neumann entropies expressed in bits unless otherwise specified. The pointer probabilities are

(7) $$p_{x}^{\Delta }\left(t\right)=\mathrm{Tr}\;\left[P_{x}{\bar{\rho }}_{\Delta }\left(t\right)\right],$$

and the temporal continuity defect is

(8) $$D_{\mathrm{cont}}\left(t;\Delta ,\tau \right)=\frac{1}{2}\sum_{x}\left|p_{x}^{\Delta }\left(t\right)-p_{x}^{\Delta }\left(t-\tau \right)\right|.$$

The reduced-state diagnostics above can also be evaluated for standard two-level open-system models, including Lindblad semigroup descriptions, even though the central model below is solved as a Hamiltonian pure-dephasing spin bath [44,45,46]. Fragment information is quantified through the Holevo quantity. It is used here as an information-theoretic benchmark and as an upper bound on accessible classical information; no fragment measurement or POVM attaining this bound is constructed. Because the Holevo quantity is nonlinear, the temporal treatment must be specified. The scalar-averaged quantity represents time-resolved trials when the classical readout-time label is retained or when the readout time is sampled and its realization remains available:

(9) $${\bar{\chi }}_{\Delta }^{\mathrm{scalar}}\left(X:F;t\right)=\int_{0}^{\Delta }w_{\Delta }\left(s\right)\chi \left(X:F;t-s\right)\;ds.$$

Equivalently, the time-tagged classical–quantum register is

(10) $$\Omega_{XFT}^{\Delta }\left(t\right)=\int_{0}^{\Delta }w_{\Delta }\left(s\right)\sum_{x}p_{x}\left(t-s\right)|x\rangle \;\langle x|⊗\rho_{F|x}\left(t-s\right)⊗|s\rangle \;\langle s|\;\;ds.$$

For a classical readout-time register *T* and time-dependent pointer priors, the complete time-tagged classical–quantum mutual information obeys the chain rule $I\left(X:FT\right)=I\left(X:T\right)+\int_{0}^{\Delta }w_{\Delta }\left(s\right)\chi \left(X:F|T=t-s\right)\;ds$. Thus, in general, the scalar average is the conditional fragment-information term and the time label can itself carry the additional classical contribution I(X:T). In the balanced QND model studied here, $p_{x}\left(t\right)=1/2$ is constant, so I(X:T)=0, and the scalar average coincides with the complete time-tagged Holevo benchmark. By contrast, the state-averaged quantity describes unresolved temporal mixing after the readout-time label has been discarded. It therefore corresponds more directly to a detector that physically integrates the conditional fragment states without preserving temporal side information. Explicitly,

(11) $$p_{x}^{\Delta }\left(t\right)=\int_{0}^{\Delta }w_{\Delta }\left(s\right)p_{x}\left(t-s\right)\;ds,$$

(12) $${\bar{\rho }}_{F|x,\Delta }\left(t\right)=\frac{\int_{0}^{\Delta }w_{\Delta }\left(s\right)p_{x}\left(t-s\right)\rho_{F|x}\left(t-s\right)\;ds}{p_{x}^{\Delta }\left(t\right)}.$$

If $p_{x}^{\Delta }\left(t\right)=0$, the corresponding conditional state has zero weight in the ensemble and may be chosen arbitrarily. We then set

(13) $$\chi_{\Delta }^{\mathrm{state}}\left(X:F;t\right)=S\;\left(\sum_{x}p_{x}^{\Delta }\left(t\right){\bar{\rho }}_{F|x,\Delta }\left(t\right)\right)-\sum_{x}p_{x}^{\Delta }\left(t\right)S\;\left({\bar{\rho }}_{F|x,\Delta }\left(t\right)\right).$$

Because these two definitions need not coincide, we also report the following conservative value in the finite-fragment exploratory comparison:

(14) $${\bar{\chi }}_{\Delta }^{\mathrm{cons}}\left(X:F;t\right)=\min\left[{\bar{\chi }}_{\Delta }^{\mathrm{scalar}}\left(X:F;t\right),\chi_{\Delta }^{\mathrm{state}}\left(X:F;t\right)\right]$$

whenever both quantities are available. This minimum never exceeds either finite-resolution construction separately. Unless otherwise stated, the scalable trajectories use ${\bar{\chi }}_{\Delta }^{\mathrm{scalar}}$ as a time-tagged benchmark. The demonstrated coexistence therefore combines the temporally integrated system channel $E_{S}^{\Delta }$, which discards the internal time label, with a fragment benchmark that retains *T*. The state-averaged and conservative quantities are finite-fragment checks of the more restrictive unresolved-time interpretation and are not used to define scalable qualifying regions.

For a bath of *N* elementary environmental spins, a fragment $F_{f}$ is a subset of *f* spins, and $H_{\Delta }\left(X;t\right)=-\sum_{x}p_{x}^{\Delta }\left(t\right)\log_{2}p_{x}^{\Delta }\left(t\right)$ is the Shannon entropy of the coarse-grained pointer distribution. In the scalable typical-fragment construction, let $f_{\delta ,\mathrm{typ}}^{\Delta }\left(t\right)$ be the smallest size for which its typical-fragment scalar-averaged Holevo benchmark satisfies

(15) $${\bar{\chi }}_{\Delta }^{\mathrm{scalar}}\left(X:F_{f}^{\mathrm{typ}};t\right)\geq \left(1-\delta \right)H_{\Delta }\left(X;t\right).$$

We define

(16) $$R_{\delta ,\mathrm{typ}}^{\Delta }\left(t\right)=\left\lfloor \frac{N}{f_{\delta ,\mathrm{typ}}^{\Delta }\left(t\right)}\right\rfloor .$$

If no size satisfies the threshold, $R_{\delta ,\mathrm{typ}}^{\Delta }\left(t\right)=0$, and the minimum size is undefined. This quantity is a typical-fragment redundancy estimate; it does not by itself construct a simultaneous partition into mutually disjoint fragments.

The sampled-fragment benchmark, $R_{\delta ,\mathrm{samp}}^{\Delta }\left(t\right)$, is defined analogously from the mean scalar-averaged Holevo information of a fixed reproducible bank of sampled subsets at each size. Each subset is drawn without replacement internally, but different draws may overlap. Thus, $R_{\delta ,\mathrm{samp}}^{\Delta }$ benchmarks average subset information and pointwise classification. It does not construct a simultaneous disjoint partition and does not validate the exact equality of the integer typical-fragment estimates. These Holevo criteria are weaker than strong Quantum Darwinism or Spectrum Broadcast Structure and should not be read as proofs of strong objectivity [11,12]. The distinction is especially important in heterogeneous environments, where non-averaged fragment information can misrepresent objectivity diagnostics [47].

Finally, define a hidden-burst index:

(17) $$I_{\mathrm{burst}}\left(t;\Delta ,\epsilon_{b},t_{\mathrm{pg}}\right)=\int_{0}^{\Delta }w_{\Delta }\left(s\right)\;1\;\left[D_{\mathrm{cl}}^{\mathrm{fine}}\left(t-s\right)>\epsilon_{b}\;\mathrm{and}\;t-s>t_{\mathrm{pg}}\right]\;ds.$$

A hidden-burst regime is present when $D_{\mathrm{cl}}\left(t;\Delta \right)$ is below a threshold while $I_{\mathrm{burst}}$ is nonzero. The choice of $t_{\mathrm{pg}}=3\;t_{\mathrm{cl}}$ is an operational cutoff used to exclude the initial Gaussian-envelope collapse; it is not a proof of non-Gaussian dynamics. Accordingly, $I_{\mathrm{burst}}$ measures late-time coherence bursts beyond that cutoff rather than residual initial coherence inside the first integration window. We write β for a prescribed lower bound on $I_{\mathrm{burst}}$, i.e., on the fraction of the detector window occupied by late-time fine-grained coherence above $\epsilon_{b}$.

**Proposition** **1** (Detector-Window Variance Decomposition)**.** *For this two-level rank-one pointer basis, let $w_{\Delta }$ be a normalized non-negative causal kernel, and let $c\left(t\right)=\rho_{01}\left(t\right)$ denote the complex off-pointer coherence amplitude. Define $D_{\mathrm{cl}}^{\mathrm{fine}}\left(t\right)=\left|c\left(t\right)\right|$ and $D_{\mathrm{cl}}^{\Delta }\left(t\right)=\left|{\langle c\rangle }_{w}\right|$, with*

(18) $${\langle c\rangle }_{w}=\int_{0}^{\Delta }w_{\Delta }\left(s\right)c\left(t-s\right)\;ds,$$

*and*

(19) $${\langle {\left|c\right|}^{2}\rangle }_{w}=\int_{0}^{\Delta }w_{\Delta }\left(s\right){\left|c\left(t-s\right)\right|}^{2}\;ds.$$

*Then,*

(20) $$D_{\mathrm{cl}}^{\Delta }\left(t\right)\leq {\langle D_{\mathrm{cl}}^{\mathrm{fine}}\rangle }_{w}$$

*and*

(21) $${\langle {\left|c\right|}^{2}\rangle }_{w}-{\left[D_{\mathrm{cl}}^{\Delta }\left(t\right)\right]}^{2}={\mathrm{Var}}_{w}\left[c\right]\left(t\right)\geq 0,$$

*where ${\mathrm{Var}}_{w}\left[c\right]\left(t\right)=\langle |c-{\langle c\rangle }_{w}{{|}^{2}\rangle }_{w}$ is the complex variance under the detector measure. Moreover,*

(22) $${\langle {\left|c\right|}^{2}\rangle }_{w}\geq \epsilon_{b}^{2}I_{\mathrm{burst}}\left(t;\Delta ,\epsilon_{b},t_{\mathrm{pg}}\right).$$

*Combining the detector-window variance identity with the burst-weight inequality gives the pointwise bound*

(23) $${\mathrm{Var}}_{w}\left[c\right]\left(t\right)\geq \epsilon_{b}^{2}I_{\mathrm{burst}}\left(t;\Delta ,\epsilon_{b},t_{\mathrm{pg}}\right)-{\left[D_{\mathrm{cl}}^{\Delta }\left(t\right)\right]}^{2}.$$

*Unlike the threshold-only bound below, this expression uses the burst weight and the coarse-grained classicality defect evaluated at the same time.*

*If, at the same time, $I_{\mathrm{burst}}\left(t;\Delta ,\epsilon_{b},t_{\mathrm{pg}}\right)\geq \beta$ and $D_{\mathrm{cl}}^{\Delta }\left(t\right)\leq \epsilon_{\mathrm{cl}}$, the pointwise bound implies the weaker threshold-only sufficient bound*

(24) $${\mathrm{Var}}_{w}\left[c\right]\left(t\right)\geq \epsilon_{b}^{2}\beta -\epsilon_{\mathrm{cl}}^{2}.$$

*This threshold-only expression may be vacuous even when the pointwise bound and the directly evaluated detector-window variance are positive.*

The proof is given in Appendix A. Evaluating this diagnostic requires access to the fine-grained trajectory inside the detector window or to a microscopic model from which that trajectory can be reconstructed; it is not inferred from the integrated system output alone. In the central-spin implementation below, the coherence amplitude is $c\left(t\right)=\Gamma_{N}\left(t\right)/2$, so $\epsilon_{b}$ thresholds $D_{\mathrm{cl}}^{\mathrm{fine}}=\left|c\right|$ and not $|\Gamma_{N}|$. We therefore report the pointwise lower bound and the directly computed detector-window variance; the threshold-only expression is retained only as a weaker sufficient statement.

**Corollary** **1** (Hidden-Burst Diagnostic)**.** *A time window is not merely decohered but hidden-burst coherent when the coarse-grained visibility $D_{\mathrm{cl}}^{\Delta }$ is below the operational threshold while the quadratic fine-grained weight ${\langle {\left|c\right|}^{2}\rangle }_{w}$ and the indicator weight $I_{\mathrm{burst}}$ remain nonzero. This model-assisted diagnostic is specific to the present two-level pinching framework because $c\left(t\right)=\rho_{01}\left(t\right)$ parameterizes the off-pointer block removed by $\Pi_{★}$.*

**Definition** **1** (Finite-Resolution Qualifying Region)**.** *Let a∈{typ,samp} identify the fragment construction. A numerically resolved grid point is a-qualified when*

(25) $$\begin{array}{cccc} D_{\mathrm{cl}}^{\Delta }\left(t\right) & \leq \epsilon_{\mathrm{cl}}, & D_{\mathrm{cont}}\left(t;\Delta ,\tau \right) & \leq \epsilon_{\mathrm{cont}},\\ R_{\delta ,a}^{\Delta }\left(t\right) & \geq R_{★}, & H_{\Delta }\left(X;t\right) & \geq H_{\min},\\ I_{\mathrm{burst}}\left(t;\Delta ,\epsilon_{b},t_{\mathrm{pg}}\right) & \geq \beta . & & \end{array}$$

Connected sequences of qualifying grid points define the numerical regions reported below. Their boundaries and durations are converged numerical estimates and should not be interpreted as an analytic proof that every continuum point satisfies all inequalities. The entropy floor $H_{\min}$ rules out a trivial stable absorbing state being misidentified as a meaningful pointer variable. In the balanced QND central-spin model below, $p_{x}^{\Delta }\left(t\right)=p_{x}\left(t\right)=1/2$, so $H_{\Delta }\left(X;t\right)=1$ bit.

Table 1 summarizes the operational notation used throughout.

The baseline parameters and checked sensitivity values are listed in Table 2.

## 3. Scalable Central-Spin Fragment-Information Model

The main physical implementation considered here is a central-spin model from spin-bath physics [21,22,23,24,25]. The model contains an explicit monitored system, many environmental spins, a dynamically selected pointer basis, and analyzable environmental records. It is nevertheless a pure-dephasing model: It does not include energy exchange, thermalization, or intra-bath scrambling. This restriction is deliberate. Recent classifications of Hamiltonians compatible with Quantum Darwinism show that pointer-compatible interaction structures matter, while interacting or dissipative baths can produce dynamics outside the exactly soluble QND setting [14,15,27]. We therefore use the model as a solvable testbed for the finite-resolution hypothesis and not as a complete microscopic model of every macroscopic environment.

The Hamiltonian is

(26) $$H_{N}=\frac{\sigma_{z}^{S}}{2}\sum_{k=1}^{N}g_{k}\sigma_{z}^{\left(k\right)},$$

with initial state

(27) $$|\Psi \left(0\right)\rangle =\frac{|0\rangle +|1\rangle }{\sqrt{2}}⊗{|+\rangle }^{⊗N}.$$

The interaction commutes with $\sigma_{z}^{S}$ and therefore selects the $\sigma_{z}$ pointer algebra dynamically. The reduced coherence of the central spin is exactly

(28) $$\rho_{01}\left(t\right)=\rho_{01}\left(0\right)\Gamma_{N}\left(t\right),\;\Gamma_{N}\left(t\right)=\prod_{k=1}^{N}\cos\left(g_{k}t\right).$$

Thus, the fine-grained classicality defect is

(29) $$D_{\mathrm{cl}}^{\mathrm{fine}}\left(t\right)=\frac{1}{2}\left|\Gamma_{N}\left(t\right)\right|$$

for the balanced initial state. Throughout the numerical analysis, $t_{\mathrm{cl}}$ denotes the short-time Gaussian-envelope estimate of the first crossing $D_{\mathrm{cl}}^{\mathrm{fine}}=\epsilon_{\mathrm{cl}}$,

$$t_{\mathrm{cl}}=\frac{1}{g_{\mathrm{rms}}}\sqrt{\frac{2\ln[1/\left(2\epsilon_{\mathrm{cl}}\right)]}{N}}.$$

It should not be confused with a later crossing determined numerically from the exact finite-bath product trajectory. The baseline late-time cutoff is $t_{\mathrm{pg}}=3\;t_{\mathrm{cl}}$. The short-time envelope follows the familiar Gaussian central-spin decay [21,22,23,24,25]. A recent theoretical preprint, used here only as supporting context, has clarified when such Gaussian central-limit behavior is valid and when it can fail in Ising spin baths [48]. The finite structured bath considered here can generate narrow recoherence bursts after the cutoff. The integrated defect $D_{\mathrm{cl}}\left(t;\Delta \right)$ is obtained from the signed coherence amplitude $\Gamma_{N}\left(t\right)$ and not from $|\Gamma_{N}\left(t\right)|$. This distinction allows positive and negative coherence amplitudes to cancel in the system channel. The scaling to N=256 does not involve a full Hilbert-space simulation of $2^{N+1}$ amplitudes: the reduced coherence is obtained from the exact product $\Gamma_{N}\left(t\right)$, while fragment Holevo quantities are estimated with the typical-fragment approximation and benchmarked against sampled subsets.

This QND structure is a strength and a limitation [14,15,21,22,23,24,25]. It makes pointer selection exact and the fragment-information calculation analytically scalable, but it also conserves pointer probabilities. Consequently, $D_{\mathrm{cont}}$ in the main central-spin model tests the stability of the pointer distribution under finite-resolution filtering and not the continuity of a changing classical trajectory. The hidden-burst claim concerns unresolved coherence for a stationary pointer distribution; it is not presented as a full model of time-dependent classical motion.

In the balanced QND baseline, $D_{\mathrm{cont}}=0$ and $H_{\Delta }\left(X;t\right)=1$ bit are analytic identities rather than independent numerical findings. The nontrivial numerical conditions are therefore the integrated classicality defect, the late-time burst weight, and the typical-fragment Holevo threshold.

To test whether the reduced-state part of the construction is destroyed by weak population dynamics, the robustness analysis includes an exact small-bath non-QND control with $H_{S}=\left(\Omega /2\right)\sigma_{y}^{S}$. For N=8, $\Omega /g_{\mathrm{rms}}$ up to 0.10 produces a nonzero pointer-population excursion up to 0.093 and $\max D_{\mathrm{cont}}$ below 0.0063, while the reduced hidden-burst window remains present. Scalable fragment redundancy is not claimed for that perturbative control; it is a robustness check on the QND idealization.

For a fragment *F* containing *f* spins, the overlap between the two conditional fragment states is

(30) $$\left|\langle E_{0}^{F}\left(t\right)|E_{1}^{F}\left(t\right)\rangle \right|=\prod_{k\in F}\left|\cos\left(g_{k}t\right)\right|.$$

For two equiprobable pointer values, the fragment Holevo information is

(31) $$\chi_{F}\left(t\right)=h_{2}\;\left(\frac{1+|\langle E_{0}^{F}\left(t\right)|E_{1}^{F}\left(t\right)\rangle |}{2}\right),$$

where $h_{2}$ is the binary entropy. Since $h_{2}\left(\left(1+q\right)/2\right)$ decreases from 1 bit to 0 as the conditional-state overlap *q* increases from 0 to 1, small fragment overlap corresponds to a high fragment Holevo quantity, matching the information-theoretic logic of Quantum Darwinism [4,5,6,7,8,10,11,12,13]. No measurement attaining this Holevo bound is constructed. In structured spin environments, the chosen fragmentation can itself determine whether a redundancy signature appears [26]. For the scalable typical-fragment calculation, we define

$$\mu \left(t\right)=\frac{1}{N}\sum_{k=1}^{N}\ln\left|\cos\left(g_{k}t\right)\right|,\;q_{f}^{\mathrm{typ}}\left(t\right)=\exp\left[f\mu \left(t\right)\right]=\exp\;\left[\frac{f}{N}\sum_{k=1}^{N}\ln\left|\cos\left(g_{k}t\right)\right|\right].$$

The corresponding time-tagged coarse-grained Holevo benchmark is

$${\bar{\chi }}_{f,\Delta }^{\mathrm{scalar}}\left(t\right)=\int_{0}^{\Delta }w_{\Delta }\left(s\right)\;h_{2}\;\left[\frac{1+q_{f}^{\mathrm{typ}}\left(t-s\right)}{2}\right]\;ds.$$

The minimal typical-fragment size $f_{\delta ,\mathrm{typ}}^{\Delta }\left(t\right)$ is the smallest *f* for which this quantity reaches $\left(1-\delta \right)H_{\Delta }\left(X;t\right)$, and the scalable estimate is $R_{\delta ,\mathrm{typ}}^{\Delta }\left(t\right)=\left\lfloor N/f_{\delta ,\mathrm{typ}}^{\Delta }\left(t\right)\right\rfloor$.

The finite-fragment scalar/state comparison now uses four fixed size-six fragments at ten points selected from the qualifying N=64 validation-grid times. Its mean scalar, state, and conservative values are 0.802, 0.732, and 0.732 bits. This exploratory comparison is reported in Appendix B; it is not used to establish scalable state-averaged redundancy.

The sampled-fragment validation was aligned with the main finite-resolution definition. For each sampled fragment, the Holevo quantity was evaluated at all midpoint nodes within the complete causal window and averaged with the same normalized boxcar kernel, $\Delta =1.20/g_{\mathrm{rms}}$, δ=0.10, and $R_{★}=4$. For each fragment size *f*, one fixed reproducible bank of 64 independently sampled subsets was generated using seed 2026 and reused across 100 validation times. Each subset was drawn without replacement internally, while different draws could overlap. The typical-fragment estimate identifies nine qualifying validation times and the sampled-mean estimate identifies eight; their pointwise classifications agree 99 out of 100 times. Exact integer estimates agree at 71%, with mean, median, P90, and maximum $|R_{\delta ,\mathrm{typ}}^{\Delta }-R_{\delta ,\mathrm{samp}}^{\Delta }|$ equal to 2.43, 0, 2.2, and 32. This is an approximate benchmark of average subset information and classification; it neither constructs a simultaneous disjoint partition nor validates the exact equality of the integer estimates [47].

The baseline numerical regions were converged before reporting. Composite midpoint calculations at $\Delta tg_{\mathrm{rms}}=0.005,0.0025,0.00125,$ and 0.000625 preserve four connected regions. Between the two finest midpoint grids, the maximum boundary displacement is $0.000625/g_{\mathrm{rms}}$, the first-time displacement is $0.000625/g_{\mathrm{rms}}$, and the relative duration change is 0.11%. On the finest grid, an independently implemented trapezoidal rule gives the same number of regions, a maximum boundary displacement of $0.000625/g_{\mathrm{rms}}$, and a 0.33% duration difference. Shifting the finest output grid by half a cell also preserves the four-region structure. The number of qualifying grid nodes changes under refinement and is therefore treated as a numerical output and not as a physical invariant.

### Numerical Parameters and Diagnostics

The baseline couplings were generated as a fixed reproducible realization of a narrow Gaussian-disorder distribution. Before rms normalization, we used

(32) $$g_{k}^{\mathrm{raw}}=1+\sigma \xi_{k}+0.35\;\sigma \sin\;\left(2\pi k\varphi \right),$$

where k=1,…,N, $\xi_{k}∼N\left(0,1\right)$ were generated with NumPy seed 123, σ=0.035, and φ=0.38196601125. The final couplings were normalized according to $g_{k}=g_{k}^{\mathrm{raw}}/\sqrt{N^{-1}\sum_{j=1}^{N}{\left(g_{j}^{\mathrm{raw}}\right)}^{2}}$ so that their rms value is unity. The deterministic quasiperiodic modulation has amplitude 0.35σ. This quasi-homogeneous realization is deliberately burst-visible and follows the finite-spin-bath motivation of central-spin decoherence and revival studies [21,22,23,24,25,48]. Broad-disorder controls are treated as negative controls, not as failed replications. The converged representative trajectory, scaling table, variance statistics, and dimensional estimate use midpoint quadrature with $\Delta tg_{\mathrm{rms}}=0.000625$. Parameter maps, ensembles, threshold controls, and finite-fragment comparisons use midpoint quadrature with $\Delta tg_{\mathrm{rms}}=0.0025$; the weak non-QND reduced-state control uses an on-grid trapezoidal filter with $\Delta tg_{\mathrm{rms}}=0.01$. Unless otherwise stated, trajectories cover $0\leq g_{\mathrm{rms}}t\leq 8$, and flags require t>Δ. Reported boundaries and grid-cell durations are numerical estimates, not continuum proofs. The burst threshold is $\epsilon_{b}=0.10$, and $t_{\mathrm{pg}}=3\;t_{\mathrm{cl}}$ is the operational cutoff excluding the initial Gaussian-envelope collapse.

**Figure 1:**
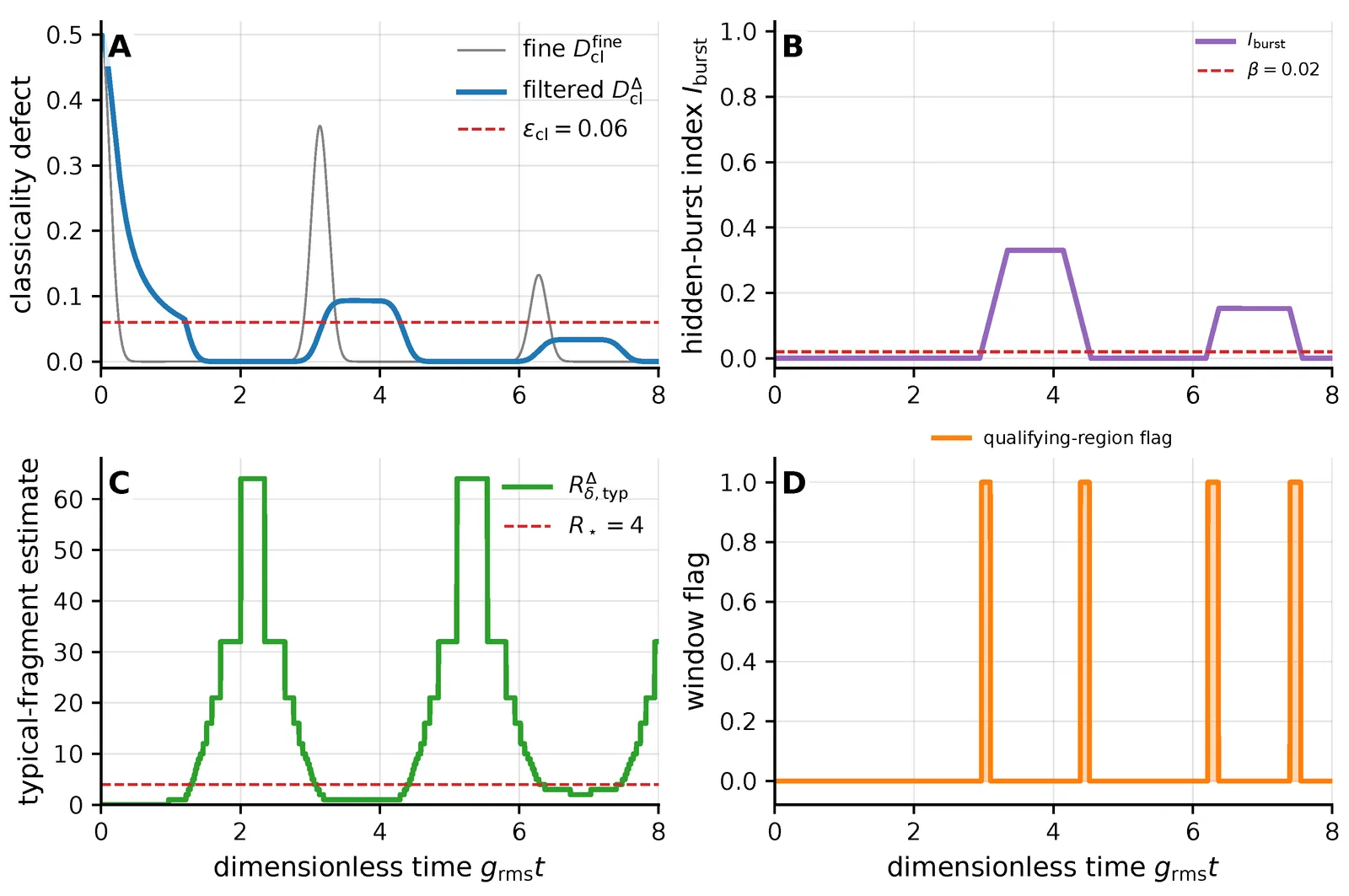
Converged central-spin trajectory for N=64. (**A**) Fine-grained classicality defect $D_{\mathrm{cl}}^{\mathrm{fine}}$ (gray), temporally integrated defect $D_{\mathrm{cl}}^{\Delta }$ (blue), and the operational threshold $\epsilon_{\mathrm{cl}}=0.06$ (red dashed). (**B**) Hidden-burst index $I_{\mathrm{burst}}$ (purple) and the threshold β=0.02 (red dashed). (**C**) Typical-fragment redundancy estimate $R_{\delta ,\mathrm{typ}}^{\Delta }$ (green) and the threshold $R_{★}=4$ (red dashed). (**D**) Numerical qualifying-region flag (orange). Fine-grained late-time bursts in $D_{\mathrm{cl}}^{\mathrm{fine}}$ are suppressed in the temporally integrated system channel, while the time-tagged fragment calculation yields the typical-fragment Holevo estimate and its corresponding numerical qualifying-region flag.

The converged representative trajectory is shown in Figure 1.

The corresponding bath-size scaling is summarized in Table 3.

**Figure 2:**
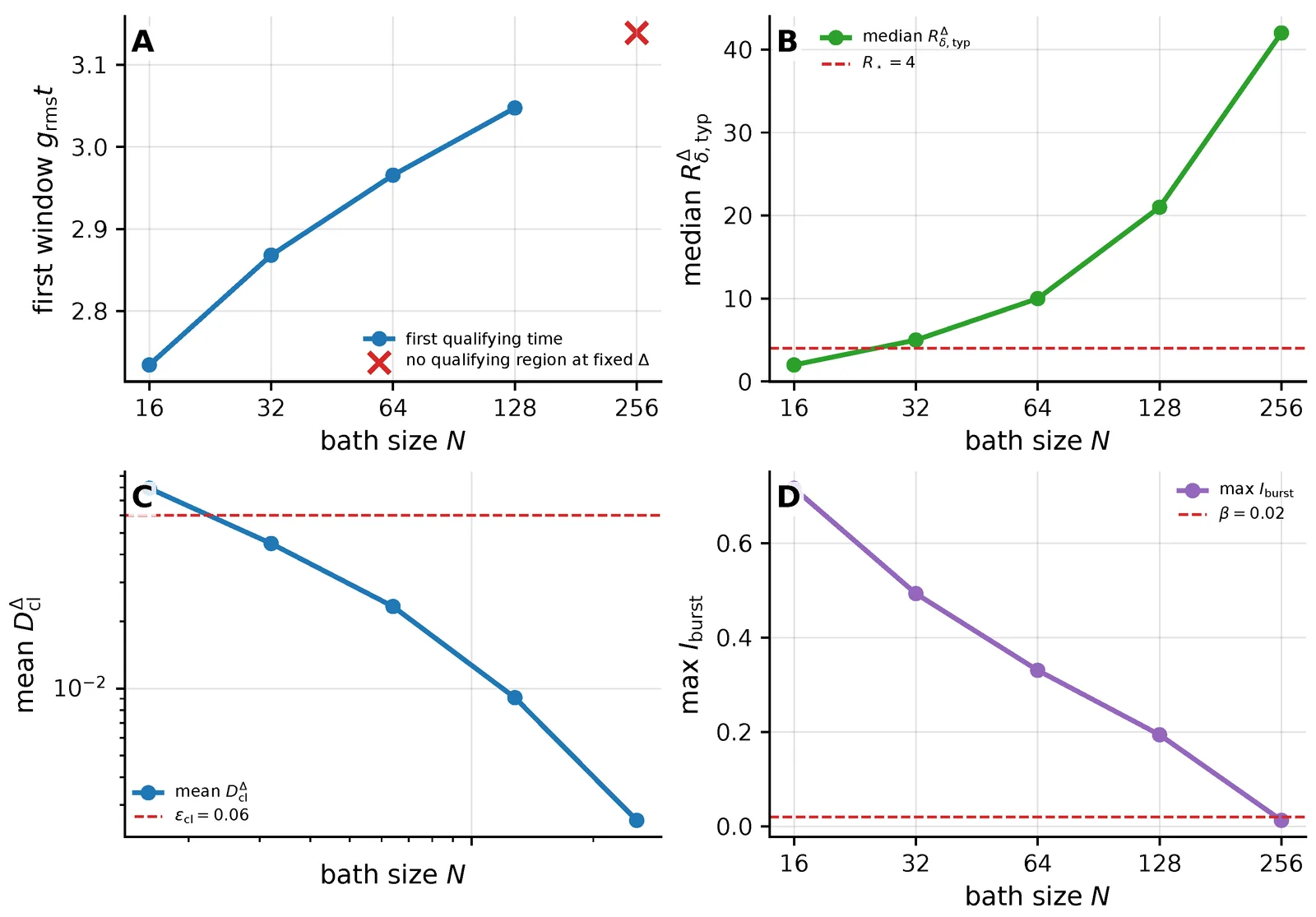
Non-monotonic finite-resolution scaling in the central-spin model. (**A**) First qualifying time as a function of bath size *N*; the red cross at N=256 indicates that no qualifying region exists at the fixed detector width Δ. (**B**) Median typical-fragment redundancy estimate $R_{\delta ,\mathrm{typ}}^{\Delta }$, with the threshold $R_{★}=4$ shown by the red dashed line. (**C**) Mean integrated classicality defect $D_{\mathrm{cl}}^{\Delta }$, with the threshold $\epsilon_{\mathrm{cl}}=0.06$ shown by the red dashed line. (**D**) Maximum hidden-burst index $I_{\mathrm{burst}}$, with the threshold β=0.02 shown by the red dashed line. Increasing *N* suppresses the mean integrated classicality defect and increases the median typical-fragment Holevo estimate, but at fixed Δ it can compress the late-time burst weight below the numerical diagnostic.

The corresponding non-monotonic finite-size trends are shown in Figure 2.

For N=64, the mean integrated classicality defect after a complete window is 0.02336, the median typical-fragment estimate is $R_{\delta ,\mathrm{typ}}^{\Delta }=10$, and the first converged qualifying time is $g_{\mathrm{rms}}t=2.9656$. The finest midpoint grid gives four numerical regions, [2.9656,3.0938], [4.3856,4.5138], [6.2131,6.3663], and [7.3938,7.5475], with total grid-cell duration $0.5656/g_{\mathrm{rms}}$. At the NV-like scale below, the individual durations are approximately 0.205–0.246μs, and the total is 0.900μs. These boundaries are converged numerical estimates and not claims about every continuum point. The threshold-only sufficient bound $\epsilon_{b}^{2}\beta -\epsilon_{\mathrm{cl}}^{2}=-3.4\times 10^{-3}$ is vacuous. The same-time pointwise bound remains positive at the numerically qualifying points; for N=64, its minimum, median, and maximum are $1.07\times 10^{-4}$, $4.09\times 10^{-4}$, and $8.25\times 10^{-4}$. The corresponding directly computed variances are $4.99\times 10^{-4}$, $1.57\times 10^{-3}$, and $6.08\times 10^{-3}$. Increasing *N* improves diagonalization and the typical-fragment estimate but can narrow late-time bursts below the diagnostic at a fixed Δ.

**Figure 3:**
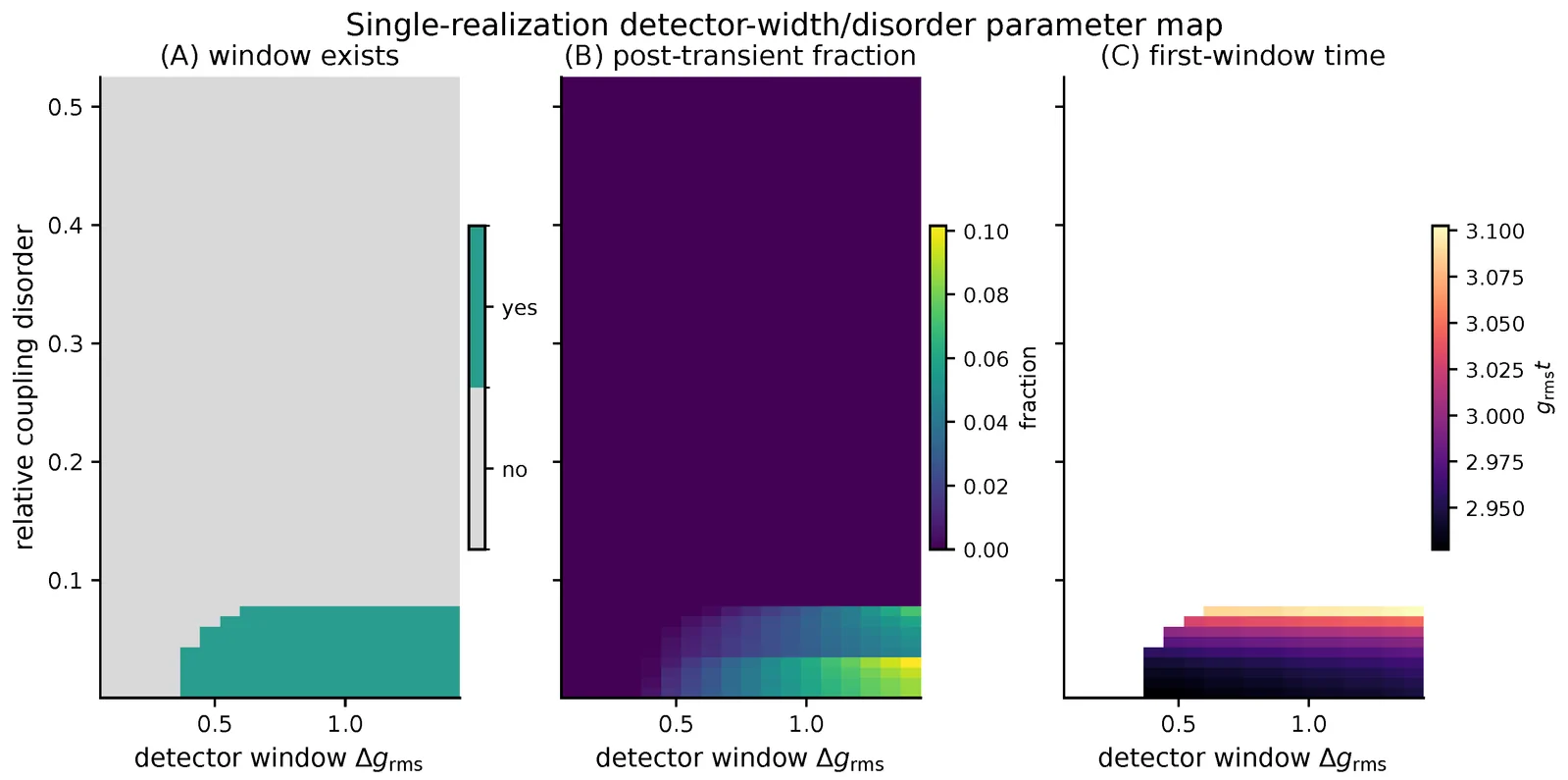
Single-realization detector-width/disorder parameter map for the fixed N=64 baseline coupling construction generated with seed 123. The panels show existence, grid fraction, and first time of a typical-fragment-qualified late-time region. This is not an ensemble-averaged phase diagram of natural NV baths; the fixed-bandwidth 100-realization ensemble is shown in Figure 4.

**Figure 4:**
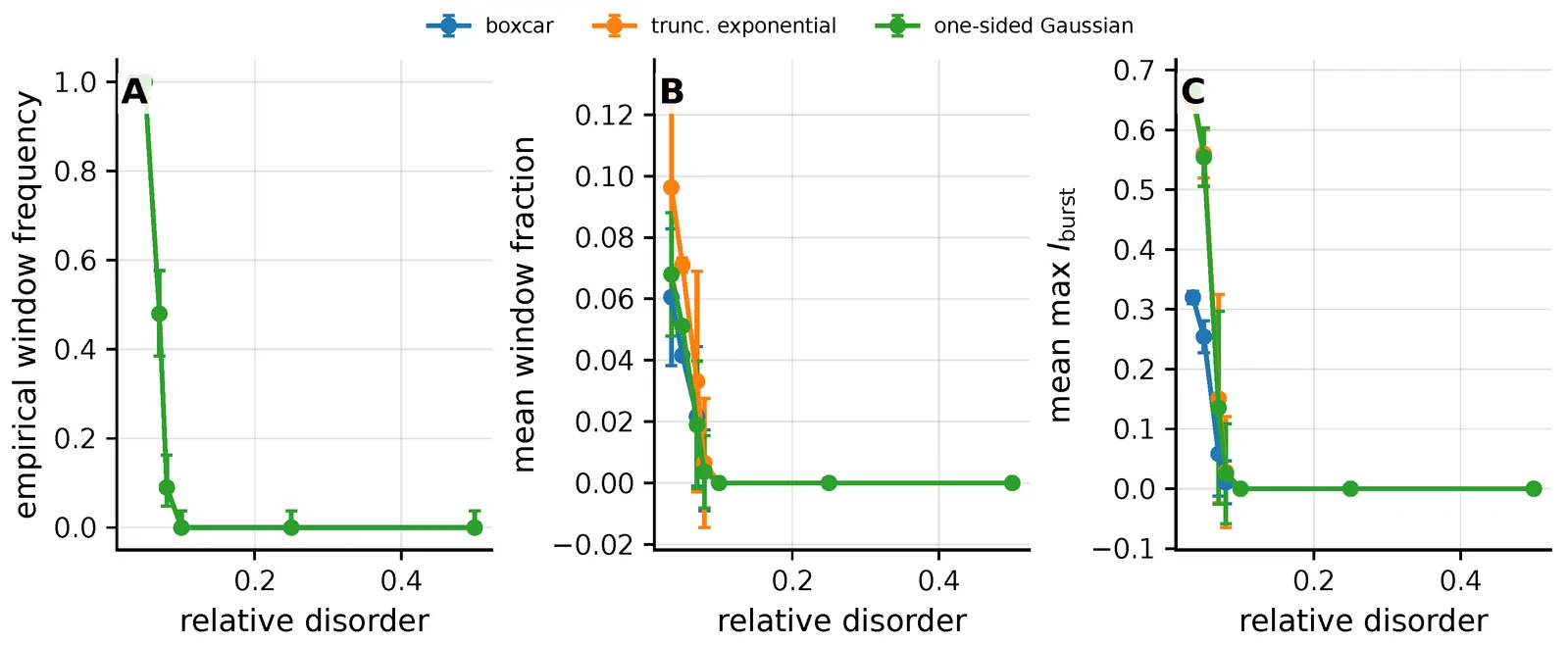
Typical-fragment disorder and detector-kernel robustness over 100 coupling realizations for N=64 at the baseline integration width. Panel (**A**) reports empirical qualifying-region frequencies with 95% Wilson intervals. Panels (**B**,**C**) show ensemble means with one-standard-deviation error bars. This synthetic ensemble is not a phase diagram of natural NV baths. In all panels, blue circles denote the boxcar kernel, orange circles the truncated exponential kernel, and green circles the one-sided Gaussian kernel.

The detector-disorder result in Figure 3 is a single-realization parameter map for the fixed baseline coupling construction generated with seed 123. It is not an ensemble-averaged phase diagram or a model of arbitrary natural NV baths. Figure 4 separately supplies a 100-realization ensemble at the baseline detector width. At fixed $\Delta =1.20/g_{\mathrm{rms}}$, N=256 has a small $D_{\mathrm{cl}}^{\Delta }$ and a high typical-fragment estimate but no qualifying late-time region. On the stated coarse detector-width grid, the separate Δ scan contains qualifying points from $\Delta g_{\mathrm{rms}}=0.40$ to 0.75. The endpoint at 0.75 is threshold-marginal at the reported numerical precision, with $\max I_{\mathrm{burst}}=\beta =0.02$; we therefore quote 0.40–0.70 as the threshold-interior range. The size scan holds the per-spin rms coupling fixed, so it is a controlled mesoscopic scan rather than a thermodynamic limit at fixed total interaction strength.

## 4. Dimensional Scale Estimate and Candidate Platforms

The central-spin model provides a dimensional reference for platforms where a controllable quantum system couples to many environmental two-level degrees of freedom. An NV-center electron spin coupled to surrounding ^13^C nuclear spins is relevant only when an engineered, quasi-homogeneous, or post-selected environment approximates the synthetic coupling structure studied here [18]. Engineered photonic or superconducting environments offer alternative settings in which fragments can be designed and characterized [19,20]. None of these citations is treated as an experimental validation of the present numerical mechanism.

The NV-like dimensional scale estimate has two parts: the short-time Gaussian-envelope crossing and the later qualifying region with time-tagged fragment Holevo information. All quoted times scale as $1/g_{\mathrm{rms}}$ when the dimensionless thresholds and synthetic coupling distribution are held fixed. In the short-time Gaussian regime,

(33) $$|\Gamma_{N}\left(t\right)|\approx \exp\;\left[-\frac{N}{2}{\left(g_{\mathrm{rms}}t\right)}^{2}\right].$$

The Gaussian-envelope crossing $t_{\mathrm{cl}}$ is the estimate defined in Section 3 and not a later crossing extracted from the exact finite-bath trajectory. The logarithm in this estimate is the natural logarithm; information entropies elsewhere are evaluated with $\log_{2}$ and reported in bits. The detector-integration window used for the hidden-burst test is $\Delta \approx 1.20/g_{\mathrm{rms}}$ in the reported parameter set. For an NV-like scale $g_{\mathrm{rms}}/\left(2\pi \right)=100\;\mathrm{kHz}$, the N=64 Gaussian crossing occurs at 0.41μs, whereas the first hidden-window time appears near 4.72μs. An experimental test should therefore not identify the short-time decay alone with the hidden-burst effect; both timescales must be resolved.

The dimensional conversion is summarized in Table 4.

A future implementation would need to prepare the central spin in a $\sigma_{x}$ superposition, reconstruct its coherence with time-resolved tomography, and separately estimate fragment information from controlled or sampled subregisters. Full fragment tomography and construction of a measurement approaching the Holevo bound are not supplied here. The reported numbers are only a dimensional scale estimate for the engineered testbed.

This two-timescale estimate is distinct from merely observing decoherence. Observing the short-time Gaussian crossing alone would test standard central-spin dephasing and not the proposed late-time mechanism. A future test would have to resolve both timescales, implement fragment-sensitive measurements, and specify whether the classical time label is retained or discarded.

## 5. Robustness Checks

Figure 4 summarizes the disorder and detector-kernel robustness tests for the typical-fragment classification. For each kernel, all 100 realizations qualify at disorder 0.035. For the boxcar kernel, the empirical frequency is 1.00 at disorder 0.05, 0.48 at 0.07, 0.09 at 0.08, and 0 at 0.10. The corresponding 95% Wilson intervals are reported in the Supplementary ensemble CSV and drawn in panel A. These synthetic controls delimit the narrow-disorder regime examined here; they do not establish robustness for arbitrary natural NV baths.

**Figure 5:**
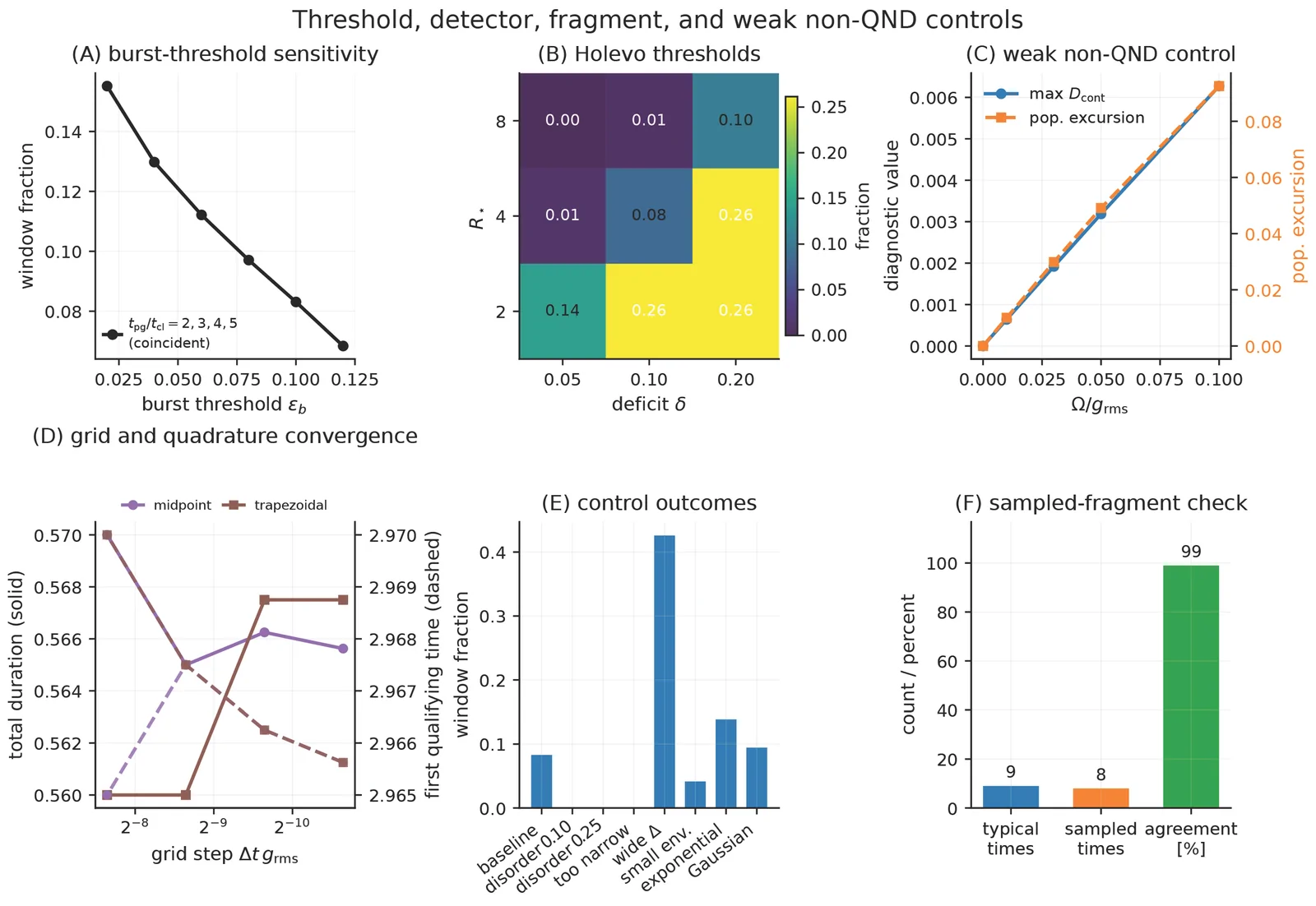
Validation and robustness controls. (**A**) Typical-fragment region fraction versus burst threshold $\epsilon_{b}$. (**B**) Sensitivity to the Holevo deficit δ and threshold $R_{★}$. (**C**) Weak non-QND reduced-state control. (**D**) Convergence of the numerically resolved qualifying regions under time-step refinement and independent midpoint/trapezoidal quadratures. Colors distinguish the two quadratures; solid curves use the left axis for the total duration, whereas dashed curves use the right axis for the first qualifying time. (**E**) Positive, negative, and bandwidth controls. (**F**) Sampled-fragment classification benchmark. In panel (**A**), the curves for $t_{\mathrm{pg}}/t_{\mathrm{cl}}=2,3,4,5$ coincide over the plotted threshold range and are therefore shown as a single curve.

Figure 5 reports sensitivity to $\epsilon_{b}$, sensitivity to the typical-fragment thresholds δ and $R_{★}$, a weak non-QND reduced-state control, grid/quadrature convergence, positive/negative/bandwidth outcomes, and sampled-fragment classification. At the robustness grid, the baseline $\epsilon_{b}=0.10$ fraction is 0.0831; increasing $\epsilon_{b}$ from 0.02 to 0.12 changes it from 0.1551 to 0.0684. All tested δ and $R_{★}$ pairs retain a typical-fragment region except δ=0.05 and $R_{★}=8$. The weak non-QND control produces pointer-population motion up to 0.093 while keeping $\max D_{\mathrm{cont}}$ below $6.3\times 10^{-3}$, but it does not test scalable fragment information. Panel D shows convergence of total duration and first qualifying time for midpoint and trapezoidal rules. The sampled-fragment classifications agree 99 out of 100 validation times.

## 6. Discussion

The manuscript evaluates a hybrid finite-resolution construction in one scalable model drawn from central-spin physics [21,22,23,24,25]. The Hamiltonian selects the pointer basis, the exact product $\Gamma_{N}\left(t\right)$ produces fine-grained recoherence bursts, and environmental subsets define fragment Holevo quantities. The result is deliberately mesoscopic and structured. Scrambling, dissipation, and non-commuting couplings require additional analysis before extrapolation [14,15,27].

Two fragment summaries must remain separate. $R_{\delta ,\mathrm{typ}}^{\Delta }$ is the scalable typical-fragment estimate, whereas $R_{\delta ,\mathrm{samp}}^{\Delta }$ benchmarks mean information over sampled subsets and their pointwise classification. Different sampled subsets may overlap. Neither quantity certifies simultaneous disjoint fragments, and the integer values in the scaling table are not interpreted as constructed partitions.

The scalable fragment result uses the scalar-averaged, time-tagged construction. The system channel instead discards its internal time label. Establishing a scalable result after applying unresolved temporal mixing to fragment states would require evaluating $\chi_{\Delta }^{\mathrm{state}}$ across sizes and throughout the numerical regions; the size-six comparison is exploratory and does not prove that claim. Moreover, the 0.9-bit threshold refers to the Holevo benchmark and must not be identified with 0.9 bits operationally extracted by a single-copy fragment measurement [4,5,6,7,8,11,12].

The hidden-burst diagnostic is model-assisted. Proposition 1 decomposes the detector-window variance but cannot reconstruct the fine trajectory from the integrated system output alone. Free-electron interferometry provides an analogy in which finer temporal and phase resolution reveals sub-cycle structure invisible to aggregated readouts [35,36]; ultrafast electron microscopy supplies a related high-resolution example [37]. The N=256 case similarly shows that a larger typical-fragment estimate need not enlarge the qualifying region at fixed bandwidth.

The remaining limitations are central. The scalable model is pure-dephasing and QND, so $D_{\mathrm{cont}}=0$ and $H_{\Delta }=1$ are identities [21,22,23,24,25]. The weak non-QND control tests only reduced-state sensitivity. The detector-width/disorder map is one fixed realization, while the ensemble is restricted to the baseline width and typical-fragment classification. Future work must address non-secular terms, bath scrambling, relaxation, measurement construction, state-integrated scaling, and stronger objectivity criteria [11,12,27].

## 7. Conclusions

This work identifies numerically converged regions in a structured QND central-spin testbed under a hybrid finite-resolution protocol. A temporally integrated system channel has low off-pointer visibility while model-resolved late-time coherence bursts remain present and a separately time-tagged scalar-averaged typical-fragment Holevo benchmark exceeds its prescribed threshold. The analysis establishes neither constructively disjoint nor state-integrated fragment redundancy.

The interaction Hamiltonian selects the pointer basis, the fine-grained central-spin coherence contains late-time finite-bath bursts, and temporal integration suppresses their system-level visibility. The fragment calculation retains a classical time label and uses the Holevo quantity only as an upper bound on accessible information; no attaining measurement is constructed. The variance diagnostic likewise presupposes the fine trajectory or a microscopic model.

The NV-like dimensional estimate places the short-time Gaussian crossing near 0.410μs and the first qualifying time near 4.720μs for N=64, $g_{\mathrm{rms}}/\left(2\pi \right)=100\;\mathrm{kHz}$, and Δ=1.91μs. These are scale-setting values for an engineered quasi-homogeneous testbed and not a direct prediction for an arbitrary natural NV bath.

The falsifiable consequence is non-universality. The numerical qualifying regions depend on integration width, bath size, coupling inhomogeneity, and thresholds. For N=64, the single-realization map establishes a lower coarse-graining boundary but not an upper-width boundary; for N=256, the sampled scan contains a threshold-interior shorter-width range together with an additional threshold-marginal coarse-grid endpoint at $\Delta g_{\mathrm{rms}}=0.75$. A too-narrow system channel resolves the bursts, while broad disorder suppresses the late-time-burst condition and therefore the full qualifying region even though the typical-fragment estimate can remain high. The result is a delimited mesoscopic mechanism with explicit failure modes and not a universal claim about classical reality.

## Figures and Tables

**Table 1:** Compact notation guide for the operational diagnostics used in this manuscript.

Symbol	Meaning	Units/Role
$\Pi_{★}$	Pinching map onto the pointer algebra	CPTP diagnostic map
$D_{\mathrm{cl}}^{\mathrm{fine}}$	Fine-grained off-pointer coherence visibility	Trace-distance scale
$D_{\mathrm{cl}}^{\Delta }$	Causally coarse-grained classicality defect	Trace distance
$D_{\mathrm{cont}}$	Temporal variation of coarse-grained pointer probabilities	Total variation distance
$R_{\delta ,\mathrm{typ}}^{\Delta }$	Typical-fragment redundancy estimate	Scalable time-tagged estimate; not a constructed partition
$R_{\delta ,\mathrm{samp}}^{\Delta }$	Sampled-fragment redundancy estimate	Sampled subsets may overlap across draws
$I_{\mathrm{burst}}$	Detector-window fraction containing late-time fine coherence above $\epsilon_{b}$	Dimensionless fraction
β	Lower bound imposed on $I_{\mathrm{burst}}$ in the hidden-burst classification	Dimensionless fraction
$R_{★}$	Prescribed minimum typical-fragment redundancy threshold	Dimensionless threshold
$\epsilon_{\mathrm{cl}},\epsilon_{\mathrm{cont}}$	Operational thresholds for classicality and continuity	Dimensionless
$\epsilon_{b}$	Fine-grained burst threshold	Trace-distance scale
$t_{\mathrm{pg}}$	Operational cutoff excluding the Gaussian-envelope collapse	Time, units $1/g_{\mathrm{rms}}$
$t_{\mathrm{cl}}$	Gaussian-envelope estimate of the first crossing $D_{\mathrm{cl}}^{\mathrm{fine}}=\epsilon_{\mathrm{cl}}$	Time, units $1/g_{\mathrm{rms}}$
Δ	Causal detector integration window	Time, units $1/g_{\mathrm{rms}}$
$g_{\mathrm{rms}}$	Root-mean-square coupling scale	Angular frequency
${\bar{\chi }}_{\Delta }^{\mathrm{scalar}}$	Time average of conditional fragment Holevo information	Time-tagged benchmark; upper bound on accessible information
$\chi_{\Delta }^{\mathrm{state}}$	Holevo information after unresolved temporal mixing of conditional fragment states	State-level finite-bandwidth diagnostic
${\bar{\chi }}_{\Delta }^{\mathrm{cons}}$	Minimum of scalar- and state-averaged Holevo diagnostics when both are computed	Exploratory finite-fragment check
$\Omega /g_{\mathrm{rms}}$	Weak transverse non-QND perturbation strength	Reduced-state robustness control
$H_{\Delta }\left(X;t\right)$	Entropy of the causally coarse-grained pointer distribution	Bits; nontrivial record floor

**Table 2:** Baseline numerical parameters and sensitivity checks. Times are expressed in units of $1/g_{\mathrm{rms}}$ unless physical units are stated explicitly.

Quantity	Baseline	Checked Values
*N*	64	16, 32, 64, 128, 256
Δ	$1.20/g_{\mathrm{rms}}$	0.10–$1.40/g_{\mathrm{rms}}$ scan; $2.50/g_{\mathrm{rms}}$ bandwidth control
τ	$0.20/g_{\mathrm{rms}}$	fixed
$\epsilon_{\mathrm{cl}}$	0.06	fixed
$\epsilon_{\mathrm{cont}}$	0.015	fixed
$\epsilon_{b}$	0.10	0.02–0.12
β	0.02	fixed
δ	0.10	0.05, 0.10, 0.20
$R_{★}$	4	2, 4, 8
$H_{\min}$	0.35 bits	fixed
$t_{\mathrm{pg}}$	$3\;t_{\mathrm{cl}}$	$2\;t_{\mathrm{cl}},\;3\;t_{\mathrm{cl}},\;4\;t_{\mathrm{cl}},\;5\;t_{\mathrm{cl}}$
disorder	0.035	0.005–0.50
kernel	boxcar	boxcar, exponential, Gaussian
quadrature	midpoint	independent trapezoidal control
Δt	$0.000625/g_{\mathrm{rms}}$	$0.005,0.0025,0.00125,0.000625/g_{\mathrm{rms}}$ convergence scan

**Table 3:** Size scaling of the central-spin model using converged midpoint quadrature. Time and Δ are in units of $1/g_{\mathrm{rms}}$. The flag requires $D_{\mathrm{cl}}^{\Delta }\leq 0.06$, $D_{\mathrm{cont}}\leq 0.015$, $R_{\delta ,\mathrm{typ}}^{\Delta }\geq 4$, $H_{\Delta }\left(X;t\right)\geq 0.35$ bits, and $I_{\mathrm{burst}}\geq \beta =0.02$. The reported mean $D_{\mathrm{cl}}^{\Delta }$ and median $R_{\delta ,\mathrm{typ}}^{\Delta }$ are trajectory summaries, not existence criteria. $R_{\delta ,\mathrm{typ}}^{\Delta }$ is a typical-fragment Holevo estimate and must not be interpreted as a constructively verified count of simultaneous disjoint fragments.

*N*	Δ	Window	First Time	Mean $D_{\mathrm{cl}}^{\Delta }$	Median $R_{\delta ,\mathrm{typ}}$	Max $I_{\mathrm{burst}}$	$t_{\mathrm{cl}}$
16	1.20	yes	2.7344	0.0793	2	0.716	0.515
32	1.20	yes	2.8681	0.0448	5	0.493	0.364
64	1.20	yes	2.9656	0.0234	10	0.331	0.257
128	1.20	yes	3.0475	0.00910	21	0.194	0.182
256	1.20	no	—	0.00256	42	0.013	0.129

**Table 4:** NV-like dimensional scale estimate for an engineered quasi-homogeneous testbed. The Gaussian crossing is an envelope estimate; the first qualifying time is obtained from the converged product dynamics. At the quoted integration window, N=256 has no qualifying region; the shorter-window scan has a threshold-interior range of approximately 0.64–1.11μs and an additional threshold-marginal coarse-grid endpoint near 1.19μs. This mapping is not a model of an arbitrary natural NV bath.

$N_{\mathrm{env}}$	$g_{\mathrm{rms}}/\left(2\pi \right)$ [Hz]	Gaussian Crossing [μs]	First Qualifying Time [μs]	Δ [μs]
16	$1.0\times 10^{5}$	0.819	4.352	1.910
32	$1.0\times 10^{5}$	0.579	4.565	1.910
64	$1.0\times 10^{5}$	0.410	4.720	1.910
128	$1.0\times 10^{5}$	0.290	4.850	1.910
256	$1.0\times 10^{5}$	0.205	—	1.910

## Data Availability

All numerical data generated in this study are provided in the Supplementary CSV Files. The custom Python 3.13.14 implementation used to generate the main figures, tables, and robustness analyses is available from the corresponding author upon reasonable request.

## Supplementary Materials

The following supporting information can be downloaded at https://www.mdpi.com/article/10.3390/e28080842/s1. The supplementary package contains the CSV data underlying the central-spin scaling analysis, converged numerical regions, midpoint/trapezoidal checks, late-time burst diagnostics, disorder and detector-kernel ensembles with Wilson intervals, typical- and sampled-fragment benchmarks, the exploratory scalar/state comparison, threshold controls, detector-window variance analysis, and the weak non-QND control.

## Appendix A. Proof of Proposition 1

Let $\;d\mu_{t}\left(s\right)=w_{\Delta }\left(s\right)\;ds$ be the normalized detector measure on [0,Δ], and define $c_{s}=c\left(t-s\right)$. Because $w_{\Delta }$ is non-negative and normalized, ${\langle c\rangle }_{w}=\int c_{s}\;d\mu_{t}\left(s\right)$ is an ordinary expectation value over the detector window.

The coarse-grained off-pointer visibility is $D_{\mathrm{cl}}^{\Delta }\left(t\right)=\left|{\langle c\rangle }_{w}\right|$, while the quadratic fine-grained weight in the same window is ${\langle {\left|c\right|}^{2}\rangle }_{w}=\int {\left|c_{s}\right|}^{2}\;d\mu_{t}\left(s\right)$. For complex $c_{s}$, the detector-window variance is ${\mathrm{Var}}_{w}\left[c\right]\left(t\right)=\langle |c-{\langle c\rangle }_{w}{{|}^{2}\rangle }_{w}$. Expanding the square gives

(A1) $${\mathrm{Var}}_{w}\left[c\right]\left(t\right)={\langle {\left|c\right|}^{2}\rangle }_{w}{-|\langle c\rangle }_{w}{|}^{2}={\langle {\left|c\right|}^{2}\rangle }_{w}-{\left[D_{\mathrm{cl}}^{\Delta }\left(t\right)\right]}^{2}\geq 0.$$

The inequality $D_{\mathrm{cl}}^{\Delta }\left(t\right)\leq {\langle D_{\mathrm{cl}}^{\mathrm{fine}}\rangle }_{w}$ follows directly from the triangle inequality, $\left|{\langle c\rangle }_{w}\right|\leq {\langle |c|\rangle }_{w}$. On the subset of the detector window where $D_{\mathrm{cl}}^{\mathrm{fine}}\left(t-s\right)=\left|c_{s}\right|>\epsilon_{b}$ and $t-s>t_{\mathrm{pg}}$, one has $|c_{s}{|}^{2}\geq \epsilon_{b}^{2}$. Therefore,

(A2) $${\langle {\left|c\right|}^{2}\rangle }_{w}\geq \epsilon_{b}^{2}I_{\mathrm{burst}}\left(t;\Delta ,\epsilon_{b},t_{\mathrm{pg}}\right).$$

Combining the two expressions at the same time gives the pointwise bound

(A3) $${\mathrm{Var}}_{w}\left[c\right]\left(t\right)\geq \epsilon_{b}^{2}I_{\mathrm{burst}}\left(t;\Delta ,\epsilon_{b},t_{\mathrm{pg}}\right)-{\left[D_{\mathrm{cl}}^{\Delta }\left(t\right)\right]}^{2}.$$

If, at that same time, $I_{\mathrm{burst}}\left(t;\Delta ,\epsilon_{b},t_{\mathrm{pg}}\right)\geq \beta$ and $D_{\mathrm{cl}}^{\Delta }\left(t\right)\leq \epsilon_{\mathrm{cl}}$, then

(A4) $${\mathrm{Var}}_{w}\left[c\right]\left(t\right)\geq \epsilon_{b}^{2}\beta -\epsilon_{\mathrm{cl}}^{2}.$$

The last expression is a weaker threshold-only sufficient bound. It may be vacuous even when the pointwise bound and the directly evaluated variance are positive. Values such as a global maximum of $I_{\mathrm{burst}}$ and a threshold value of $D_{\mathrm{cl}}^{\Delta }$ cannot be combined unless they refer to the same time. The Supplementary pointwise CSV and its window-restricted summary therefore evaluate all terms on identical temporal indices.

## Appendix B. Holevo Coarse-Graining and Finite-Fragment Benchmarks

We distinguish scalar-averaged Holevo information, ${\bar{\chi }}_{\Delta }^{\mathrm{scalar}}=\int w_{\Delta }\chi \left(t-s\right)\;ds$, from the state-averaged quantity obtained by first averaging conditional fragment states and then computing χ. The scalar quantity retains a classical time label; the state-averaged quantity discards it. Both remain Holevo benchmarks, and no attaining measurement is constructed. The normalized conditional state is

(A5) $${\bar{\rho }}_{F|x,\Delta }\left(t\right)=\frac{\int w_{\Delta }\left(s\right)p_{x}\left(t-s\right)\rho_{F|x}\left(t-s\right)\;ds}{p_{x}^{\Delta }\left(t\right)}.$$

Because χ is nonlinear, the two quantities need not agree. The conservative diagnostic ${\bar{\chi }}_{\Delta }^{\mathrm{cons}}=\min\left({\bar{\chi }}_{\Delta }^{\mathrm{scalar}},\chi_{\Delta }^{\mathrm{state}}\right)$ is reported only as a finite-fragment exploratory check. We used one fixed bank of four size-six fragments (seed 4242), reused at ten qualifying control times on the $0.0025/g_{\mathrm{rms}}$ grid. This calculation is not used to establish the scalable numerical regions. The finite-fragment comparison is summarized in Table A1.

**Table A1:** Exploratory finite-fragment comparison of scalar- and state-averaged Holevo quantities at ten selected control times. Standard deviations are over 40 paired time–fragment cases. This analysis is not used to establish the scalable qualifying regions.

Construction	Mean [Bits]	Standard Deviation [Bits]	Median [Bits]
Time-tagged scalar average	0.802	0.058	0.809
State average after discarding time	0.732	0.082	0.745
Conservative minimum	0.732	0.082	0.745

For the sampled-fragment benchmark, the Holevo quantity was evaluated at all midpoint nodes of each complete causal window and averaged with the normalized kernel. We used the same boxcar kernel, $\Delta =1.20/g_{\mathrm{rms}}$, δ=0.10, $R_{★}=4$, and β=0.02. One fixed bank of 64 subsets per size was generated with seed 2026 and reused across 100 validation times. Each subset was sampled without replacement internally, while different draws could overlap. The typical and sampled estimates identify nine and eight qualifying times and agree in classification 99 out of 100 times. Exact integer estimates agree in 71%; the mean, median, P90, and maximum absolute differences are 2.43, 0, 2.2, and 32. This is an approximate subset benchmark, not a disjoint-partition calculation.

The convergence tables report midpoint and trapezoidal results on four nested grids. The predeclared checks require the same number of connected regions, maximum boundary and first-time shifts no larger than $0.0025/g_{\mathrm{rms}}$, and a relative duration change no larger than 1%. The two finest midpoint grids and the finest midpoint/trapezoidal comparison pass all criteria. A half-cell offset control also preserves the region structure. The public CSVs report grid-dependent node counts only as numerical diagnostics.

## Appendix C. Detector Kernels, Late-Time Cutoff, and Threshold Sensitivity

The main figures use a boxcar detector kernel, $w_{\Delta }\left(s\right)=1/\Delta$ on 0≤s≤Δ. Robustness checks use the normalized truncated exponential kernel

(A6) $$w_{\Delta }^{\exp}\left(s\right)=\frac{e^{-s/\tau_{k}}}{\tau_{k}\left(1-e^{-\Delta /\tau_{k}}\right)},\;\tau_{k}=\frac{\Delta }{3},\;0\leq s\leq \Delta ,$$

and the normalized one-sided Gaussian kernel

(A7) $$w_{\Delta }^{G}\left(s\right)=\frac{e^{-s^{2}/\left(2\sigma_{k}^{2}\right)}}{\sigma_{k}\sqrt{\pi /2}\;\mathrm{erf}\;\left(\frac{\Delta }{\sqrt{2}\sigma_{k}}\right)},\;\sigma_{k}=\frac{\Delta }{3},\;0\leq s\leq \Delta .$$

In production, each kernel is evaluated at midpoint nodes and normalized by its discrete weighted sum; the trapezoidal control is implemented independently on both endpoints and interior nodes. The cutoff is $t_{\mathrm{pg}}=3\;t_{\mathrm{cl}}$ in the main tables. Scans over $t_{\mathrm{pg}}/t_{\mathrm{cl}}=2,3,4,5$ and $\epsilon_{b}=0.02$–0.12 preserve a typical-fragment qualifying region in the quasi-homogeneous N=64 bath. At $\epsilon_{b}=0.10$, the robustness-grid fraction is 0.0831; varying $\epsilon_{b}$ from 0.02 to 0.12 changes it from 0.1551 to 0.0684. A separate scan over δ=0.05,0.10,0.20 and $R_{★}=2,4,8$ preserves a region except at δ=0.05, $R_{★}=8$.

## Appendix D. Weak Non-QND Control and Disorder/Bandwidth Controls

To test the QND idealization, we added $H_{S}=\left(\Omega /2\right)\sigma_{y}^{S}$ and solved a finite N=8 bath exactly. For $\Omega /g_{\mathrm{rms}}=0.01,0.03,0.05,0.10$, the maximum pointer-population excursions are approximately 0.010, 0.030, 0.049, and 0.093, while $\max D_{\mathrm{cont}}<0.0063$ and a reduced-state signature persists. This does not prove scalable non-QND objectivity. Broad disorder levels of 0.10 and 0.25 eliminate the full qualifying region at $\Delta =1.20/g_{\mathrm{rms}}$ because the late-time burst index falls to zero, even though the typical-fragment estimate itself remains high; a too-narrow integration window resolves rather than hides the fine bursts. These controls delimit the synthetic mesoscopic regime.
